# Mechanochemical Solvent-Free Synthesis of Quaternary Semiconductor Cu-Fe-Sn-S Nanocrystals

**DOI:** 10.1186/s11671-017-2029-5

**Published:** 2017-04-05

**Authors:** Peter Baláž, Matej Baláž, María J. Sayagués, Ivan Škorvánek, Anna Zorkovská, Erika Dutková, Jaroslav Briančin, Jaroslav Kováč, Jaroslav Kováč, Yaroslav Shpotyuk

**Affiliations:** 1 Institute of Geotechnics, Slovak Academy of Sciences, Košice, 04001 Slovakia; 2 Institute of Materials Science of Seville, CSIC-US, Seville, 41092 Spain; 3 Institute of Experimental Physics, Slovak Academy of Sciences, Košice, 04001 Slovakia; 4 Institute of Electronics and Photonics, Slovak University of Technology, Bratislava, 81219 Slovakia; 5 Department of Sensor and Semiconductor Electronics, Ivan Franko National University of Lviv, 107, Tarnavskogo str., Lviv, 79017 Ukraine; 6 Center for Innovation and Transfer of Natural Sciences and Engineering Knowledge, Faculty of Mathematics and Natural Sciences, University of Rzeszow, 1, Pigonia str., Rzeszow, 35-958 Poland

**Keywords:** Stannite, Mechanochemistry, Quaternary sulfide, Solar cell

## Abstract

In this study, we demonstrate a one-pot mechanochemical synthesis of the nanocomposite composed of stannite Cu_2_FeSnS_4_ and rhodostannite Cu_2_FeSn_3_S_8_ nanocrystals using a planetary ball mill and elemental precursors (Cu, Fe, Sn, S). By this approach, unique nanostructures with interesting properties can be obtained. Methods of XRD, Raman spectroscopy, UV-Vis, nitrogen adsorption, SEM, EDX, HRTEM, STEM, and SQUID magnetometry were applied. Quaternary tetragonal phases of stannite and rhodostannite with crystallite sizes 18–19 nm were obtained. The dominant Raman peaks corresponding to the tetragonal stannite structure corresponding to A-symmetry optical modes were identified in the spectra. The bandgap 1.25 eV calculated from UV-Vis absorption spectrum is very well-acceptable value for the application of the synthesized material. The SEM micrographs illustrate the clusters of particles in micron and submicron range. The formation of agglomerates is also illustrated on the TEM micrographs. Weak ferromagnetic properties of the synthesized nanocrystals were documented.

## Background

Nanocrystalline quaternary chalcogenides possess several advantages when compared with their bulk analogs, namely the tunability of bandgap and good control of the internal structure and deposition procedures [1]. However, there is a general paradox in the present research and the application of quaternary chalcogenide materials for solar cells. On the one side, CuIn_1 − *x*_Ga_*x*_Se_2_ (CIGS) thin film solar cells attracted a big attention owing to their high power conversion efficiency (21.7%) and good stability. On the other side, these materials represent a potential environmental problem because of selenium toxicity, indium and gallium limited availability, and high price [2–6]. Kesterite Cu_2_ZnSnS_4_ (CZTS) and stannite Cu_2_FeSnS_4_ (CFTS) provide promising alternatives to CIGS, because of their environmental acceptance (application of sulfur instead of selenium) and economic advantages (application of earth-abundant iron, zinc, and tin instead of scarce indium and gallium). However, in comparison with CIGS, their power conversion efficiency is lower (12.6 and 7.2% for CZTS and CFTS, respectively [2, 3, 7]). There are several “classical” methods to synthesize CZTS and CFTS nanocrystals, like electrochemical, vacuum and thermal deposition, electron beam evaporation, magnetron sputtering, spray pyrolysis, pulse laser deposition, and sol-gel method [5, 8]. Besides them, other methods like microwave treatment [9, 10], solvothermal synthesis [11–14], hot-injection [15–17], electrospinning [18], and dip-coating [19] were successfully applied. However, these techniques are complex and time-consuming and need high temperature, and in some cases also the utilization of toxic organic solvents is necessary.

Solid-state synthesis methods offer several advantages. Very frequently, the synthesis course is accelerated by the application of mechanochemistry, where the high-energy milling is being applied to induce and speed up chemical reactions [20–22]. This approach is simple, solvent-free, and reproducible, and the synthesis might be easily scaled up. There are only several reports on mechanochemical synthesis of CZTS(Se) nanoparticles during which no solvents or additives were used [8, 23–35]. To the best of our knowledge, there have been no reports on the synthesis of stannite Cu_2_FeSnS_4_ by mechanochemical approach until now.

## Methods

### Starting Materials

The starting materials, namely elemental Cu (99%, Merck, Germany), Fe (99%, Merck, Germany), Sn (99.9%, Merck, Germany), and S (99%, CG-Chemikalien, GmbH, Germany), were weighed and mixed in atomic ratios of 2:1:1:4 (according to the stoichiometry Cu_2_FeSnS_4_). The mixture was subjected to high-energy milling, as described in the “Synthesis” section.

### Synthesis

The mechanochemical synthesis has been performed in a planetary ball mill Pulverisette 6 (Fristsch, Germany) working under the following conditions: loading—50 balls (*d* = 10 mm) of tungsten carbide (WC), milling pot volume—250 mL, milling pot material—WC, total weight of reactants—5 g, ball-to-powder mass ratio—70, milling speed—500 min^−1^, milling time—60–120 min, and milling atmosphere—argon.

### Characterization Methods

The crystal structure was characterized using a D8 Advance Bruker X-ray diffractometer in the Bragg-Brentano geometry, working with a CuKα (*λ* = 0.15418 nm) radiation and a scintillation detector. The data were collected over the angular range 10° < 2 theta < 100° with scanning steps of 0.02° and the measurement step time interval of 10 s. For the data processing, the commercial Bruker tools have been used. Namely, for the phase identification, the Diffrac^plus^ Eva and the ICDD PDF-2 database and, for Rietveld analysis, the Diffrac^plus^ Topas software have been applied.

Micro-Raman spectra were collected in air at room temperature, focusing the beam of Ar laser line (514 nm) via a confocal Raman microscope (Spectroscopy&Imaging, Germany) utilizing system in a backscattering geometry with a spot diameter size ~2 μm. This ensures the excitation of few randomly oriented microcrystalline grains in the powder sample. To minimize the thermal effect on the sample, the excitation power during Raman spectra measurements was changed; concretely, different attenuation 20 and 50% of the maximum laser power (80 mW) was used. The recrystallization of the microcrystalline grains was observed after measurements at the 50% of maximum laser power. The frequency of the Raman line of a crystalline Si at 520 cm^−1^ was used to calibrate the measuring system.

Optical studies were carried out using a UV-Vis spectrophotometer Helios Gamma (Thermo Electron Corporation, Great Britain) in a quartz cell by dispersing the synthesized CFTS particles in absolute ethanol by ultrasonic treatment.

A nitrogen-adsorption apparatus NOVA 1200e Surface Area & Pore Size Analyzer (Quantachrome Instruments, Great Britain) was employed to record the nitrogen adsorption-desorption isotherms at the temperature of liquid nitrogen. The specific surface area (*S*_BET_) values were calculated using the Brunauer-Emmett-Teller (BET) equation. The pore size distribution was calculated using the Barrett-Joyner-Halenda (BJH) method.

The morphology and the structure at the microscopic level were analyzed using a scanning electron microscopy (SEM). A small quantity of the CFTS sample was deposited on carbon-coated copper grids to avoid the interference between the Cu grid and the Cu from the sample in the energy-dispersive X-ray (EDX) analysis. A MIRA 3 FE-SEM microscope (TESCAN, Czech Republic) equipped with an EDX detector (Oxford Instruments, UK) was used. The sample was sufficiently conductive; thus, it was not necessary to coat it with a conductive material in order to avoid charging artifacts.

A FEG high-resolution transmission electron microscope (HRTEM) from FEI Company, USA (model TECNAI G2 F30 S-twin), with scanning transmission capabilities (STEM) was also used. The experiments were conducted at 300 kV with 0.2-nm point resolution. The microscope is equipped with a high-angle annular dark-field (HAADF) detector from Fischione Instruments, USA (0.16-nm point resolution), and an INBCA ZX-max 80 silicon drift detector for the EDX analysis. The GHR micrograph analysis, lattice spacing, fast Fourier transform (FFT), and phase interpretation were done with the Gatan Digital micrograph software (Gatan Inc., USA) and the Java version of the Electron Microscope Software (JEM). The analysis of the HAADF-STEM images and the EDX spectra profile were conducted with the ES Vision software (FEI Company, USA).

The measurements of the DC magnetization as a function of temperature and applied field were performed by Magnetic Property Measuring System model MPMS-XL-5 (Quantum Design, USA) equipped with a 5-T superconducting magnet. The zero-field-cooled and field-cooled magnetization data were collected in the temperature range from 2 to 300 K. The magnetization curves as a function of applied field were obtained at 5 and 300 K. The sequence of measurements was as follows: (i) the sample was cooled to 2 K in zero field, and after the application of external field of 10^3^ or 10^4^ Oe, the zero-field-cooled (ZFC) magnetization was determined during warming to 300 K; (ii) the field-cooled (FC) curves were recorded during the cooling from 300 K down to 2 K in the same external field.

## Results and Discussion

### Structural Analysis

**Fig. 1:**
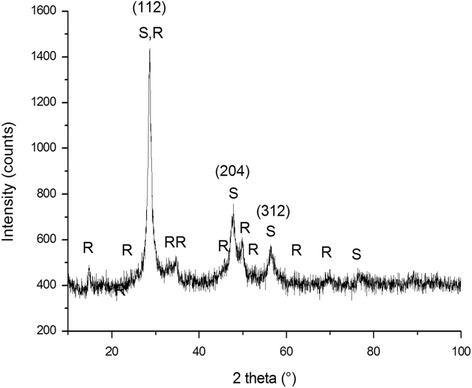
XRD pattern of the CFTS sample: *R* rhodostannite Cu_2_FeSn_3_S_8_, *S* stannite Cu_2_FeSnS_4_

The XRD pattern of the elemental mixture (Cu, Fe, Sn, and S powders) milled for 120 min is shown in Fig. 1. Peaks corresponding to the starting elements are not present, and all the observed ones can be assigned to tetragonal (scalenohedral) body-centered stannite Cu_2_FeSnS_4_ (JCPDS 44-1476) and tetragonal (dipyramidal) body-centered rhodostannite Cu_2_FeSn_3_S_8_ (JCPDS 85-0378). This is in contradiction with the observation of Bernardini et al., where, by thermal synthesis, also, the minor traces of herzenbergite SnS besides stannite have been detected [36, 37]. However, the quaternary Cu-Fe-Sn-S system is more complex, and besides the mentioned tetragonal forms, also, cubic Cu_2_FeSnS_4_ (isostannite) and hexagonal Cu_2_Fe_2_SnS_6_ (hexastannite) can exist [38]. In our case, no peaks confirming the presence of other crystallographic forms could be observed. The XRD pattern exhibits three most intensive peaks at 2*θ* = 28.5°, 47.5°, and 56.0°, which can be assigned to the (112), (204), and (312) planes of the tetragonal crystals (Fig. 1).

The Rietveld fitting method has been applied for the analysis of the experimental XRD data. Semi-quantitative analysis revealed that the main phase component is stannite with 76% of weight content, while only 24% of rhodostannite content was estimated. The microstructure data are summarized in Table 1. The determined cell volume for stannite, *V*_S_ = 313.74 Å^3^, is by 1.5% smaller than the database value (318.99 Å^3^). The strain, *ε*_S_ = 0.73%, is quite large, as expected for mechanosynthesized materials with strongly distorted structures. The rhodostannite cell volume, *V*_RS_ = 553.46 Å^3^, is by 0.4% larger than the database value, 551.24 Å^3^, and the strain is *ε*_RS_ = 0.41%. The calculated crystallite sizes are 18.5 and 19 nm for stannite and rhodostannite, respectively. The values are very close to size 16.6 nm reported in [39], where the films of Cu_2_FeSnS_4_ were synthesized. The relationship regarding stannite (S) and rhodostannite (R) transformation can be hypothesized as follows: in nature, (R) is a replacement (alteration) product of (S) [40, 41]. This alteration proceeds for a long time and usually under high pressures and temperatures. In analogy, the same transformation can be achieved by the application of high-energy milling, where high local pressures and contact temperatures exist [22]. However, the reaction period is substantially shortened. We speculate that during milling, gaseous SnS and S_2_ are liberated from (R) and mutual coexistence of both phases can be explained according to the hypothetical equation:

**Table 1:** Results obtained from Rietveld analysis of XRD data for the CFTS sample

Parameter	Stannite Cu_2_FeSnS_4_	Rhodostannite Cu_2_FeSn_3_S_8_
Space group	I-42m	I-41/a
Crystallite size (nm)	18.5	19
Strain, *ε* (%)	0.73	0.41
Lattice parameters		
a (Å)	5.4029 ± 1E−3	7.3011 ± 1.5E−3
c (Å)	10.7471 ± 3E−3	10.3828 ± 3E−3
Cell volume, *V* (Å^3^)	313.74	553.46
Occurrence (%)	76	24

1 $$ {\mathrm{Cu}}_2{\mathrm{FeSn}}_3{\mathrm{S}}_8-2\left(\mathrm{SnS}+\mathrm{S}\right)\leftrightarrow {\mathrm{Cu}}_2{\mathrm{FeSn}\mathrm{S}}_4 $$

### Optical Properties

#### Raman Spectroscopy

In comparison with X-ray diffractometry, Raman spectroscopy provides more detailed information about the crystalline structure and its purity [42, 43]. As stated in [17], X-ray diffraction peaks of the quaternary Cu_2_FeSnS_4_ are very similar to FeS and Cu_3_SnS_4_, and therefore, more appropriate method is necessary for its clear determination. Raman spectroscopy can be a proper tool.

**Fig. 2:**
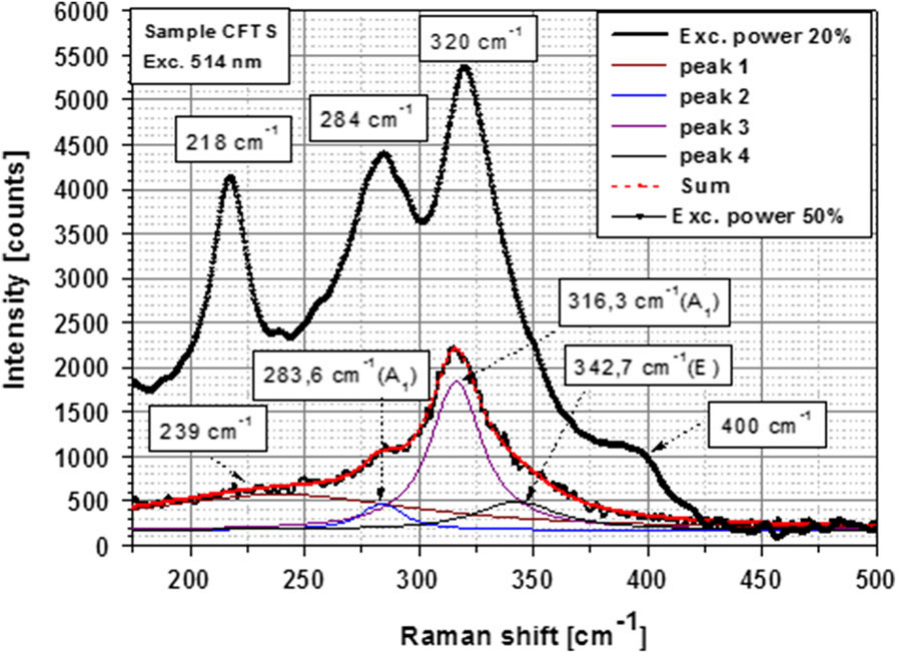
Raman spectrum measured at 20 and 50% excitation maximum laser power and fitting of the spectrum (20%) with the Lorentzian curves

In Fig. 2, the Raman spectrum of CFTS sample measured at 20 and 50% of maximum laser power shows a set of peaks in the range of 175–500 cm^−1^. The fitting of the spectrum (20%) with the Lorentzian curves has allowed the identification of four peaks at 239, 283.6, 316.3, and 342.7 cm^−1^ corresponding to stannite structure (I-42m space group). Among them, the two dominant peaks at 283.6 and 316.3 cm^−1^ are practically identical with the spectra of the tetragonal Cu_2_FeSnS_4_ assigned to A-symmetry optical modes as prepared by the crystal growth process and described in [17, 42]. The long tails of Raman spectrum in the range of 175–260 and 320–400 cm^−1^ include few weak peaks, fitted by broad peak at 239 cm^−1^, as well as the peak at 342.7 cm^−1^. These peaks could be assigned to the presence of 24% rhodostannite phase in the powder, as estimated by XRD measurements. By comparing the Raman spectrum of stannite and rhodostannite from the RRUFF database, Raman spectrum of rhodostannite approximately overlaps with stannite spectrum, showing weaker and broader peaks in the range of 250–400 cm^−1^. The confirmation of the stannite tetragonal structure from Raman measurements was also documented in papers [6, 11, 15]. In addition, Raman peaks corresponding to the tetragonal structure of space group P4 were detected at 218, 284, 320, and 400 cm^−1^ after thermal recrystallization of the microcrystalline grains in the sample measured at 50% of maximum laser excitation power (visible change under optical microscope). This is in good agreement with the paper published by Rincon et.al [38] and confirmed the possible thermal recrystallization of the powder under strong laser beam.

#### UV-Vis Spectroscopy

**Fig. 3:**
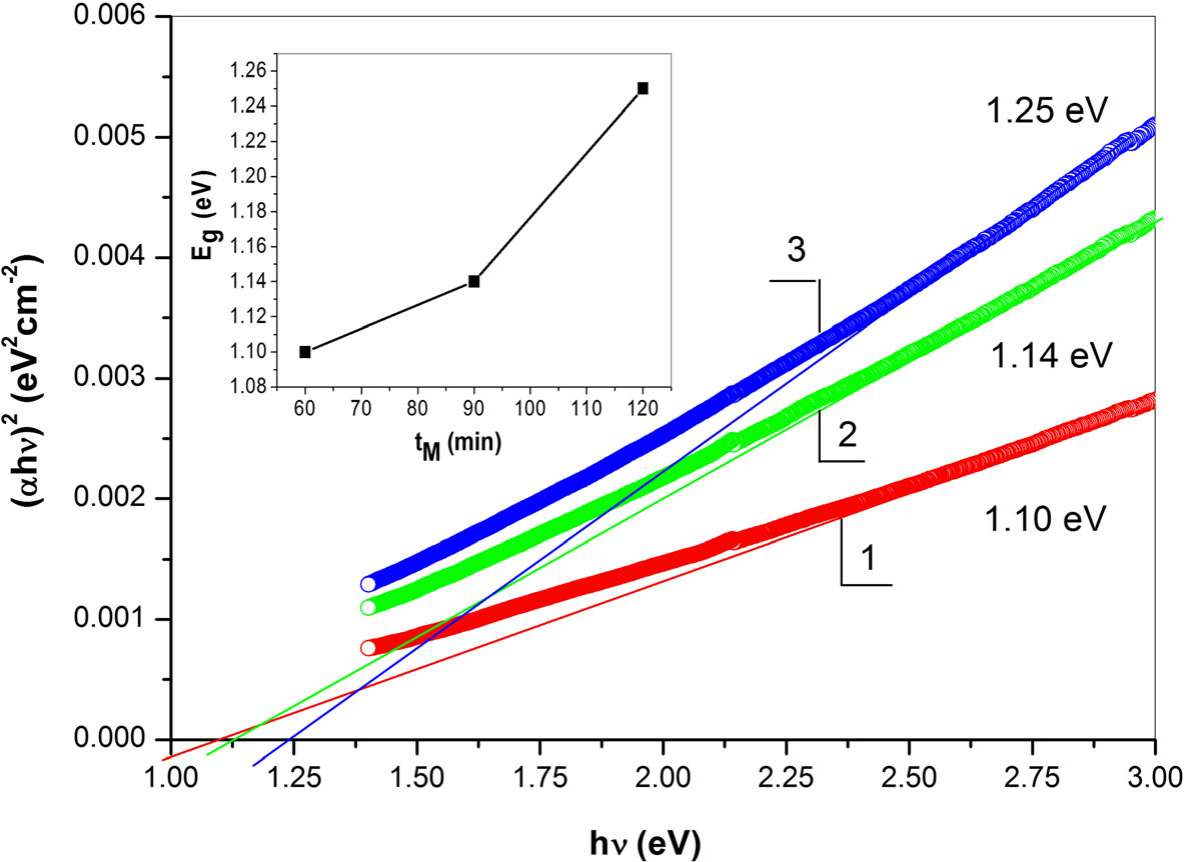
Optical bandgap estimation for CFTS samples synthesized by milling at 60 (1), 90 (2), and 120 (3) min (the dependence on milling time is given in the *inset*)

The optical properties of the CFTS sample were investigated using UV-Vis absorption spectroscopy. Figure 3 shows the corresponding spectrum which exhibits a broad absorption in the whole visible region. Similar spectra have been reported in several papers dealing with the optical properties of the CFTS [8, 14, 16, 18, 23]. The bandgap energy of the CFTS sample was determined by plotting the square of absorption coefficient (*α*) times photon energy (*h*ν) vs. *h*ν, according to the formula *αhν* = *α*(*hν* − *E*_g_)^*n*/2^, where *α*, *h*, *ν*, *E*_g_ and *A* are the optical absorption coefficient, Planck’s constant, light frequency, bandgap energy, and a constant, respectively [9]. By extrapolating (*αhν*)^2^ vs. *h*ν plot to (*αhν*)^2^ = 0 (inset), its bandgap can be calculated.

In the present case, the optical bandgaps determined from UV-Vis spectra are 1.10, 1.14, and 1.25 eV for the samples milled for 60, 90, and 120 min, respectively. The values are very well-acceptable for the application of CFTS as photo-absorbers in solar cell materials [6, 8–10, 14, 15, 17, 18, 39, 44]. The inset in Fig. 3 illustrates the possibility to manipulate the values of *E*_g_ as a consequence of milling. The increase in *E*_g_ is a consequence of the particle size reduction during milling. This new phenomenon broadens the possibility of bandgap tuning in CFTS not only by chemical means (e.g., by variation of thermal annealing and/or composition) but also by mechanical ones (e.g., by milling) [20].

### Surface Properties

**Fig. 4:**
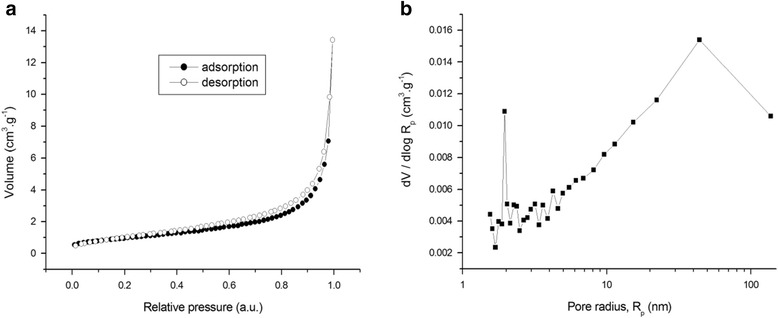
Surface properties of the CFTS sample. **a** Nitrogen adsorption/desorption isotherm. **b** Pore size distribution

The surface properties of the mechanochemically prepared CFTS were investigated by measuring the whole adsorption-desorption isotherm of the studied sample and the calculation of pore size distribution from the desorption curve. The results are presented in Fig. 4.

The value of the specific surface area of the studied sample is 3.5 m^2^ g^−1^, which is significantly lower compared to the value 14.4 m^2^ g^−1^ reported in [9]. The difference is quite understandable, as in the present work, dry milling of the elemental precursors was used, where the impacts of the milling balls on the milled powder lead to the destruction of possible porous cavities. On the other hand, in the cited work [9], the solution-based approach and microwave irradiation was used, where the porous structure could freely develop. From the adsorption-desorption isotherms of the CFTS sample prepared in this work (Fig. 4a), it can be seen that the porous properties are not very rich, as there is not a big difference between the adsorption and desorption curve. As there is no significant hysteresis loop, the number of mesopores should also be not so high. The present isotherm seems to be of type II, according to classification proposed by Singh et al. [45], which is characteristic for non-porous and macroporous adsorbents. The shape of the isotherms in the area of the highest relative pressure hints to the large amount of macropores. The results of the pore size distribution measurements (Fig. 4b) confirm the fact that there is not a large amount of mesopores in the sample and that it is mainly macroporous. There is a small local maximum in the area around 2 nm, but this is an artifact, as it was not present when the pore size distribution was calculated from the adsorption curve (not shown here). The presented results document poorer pore properties comparing to the work [9], where mainly mesoporous structure with the traces of macropores was reported.

To summarize the surface properties, the formation of macroporous stannite was achieved and the general phenomenon of dry milling, namely the formation of the compounds at high pressures and temperatures generally leading to the formation of the agglomerates with low specific surface area [46], was confirmed for this case.

### Microstructural Analysis

**Fig. 5:**
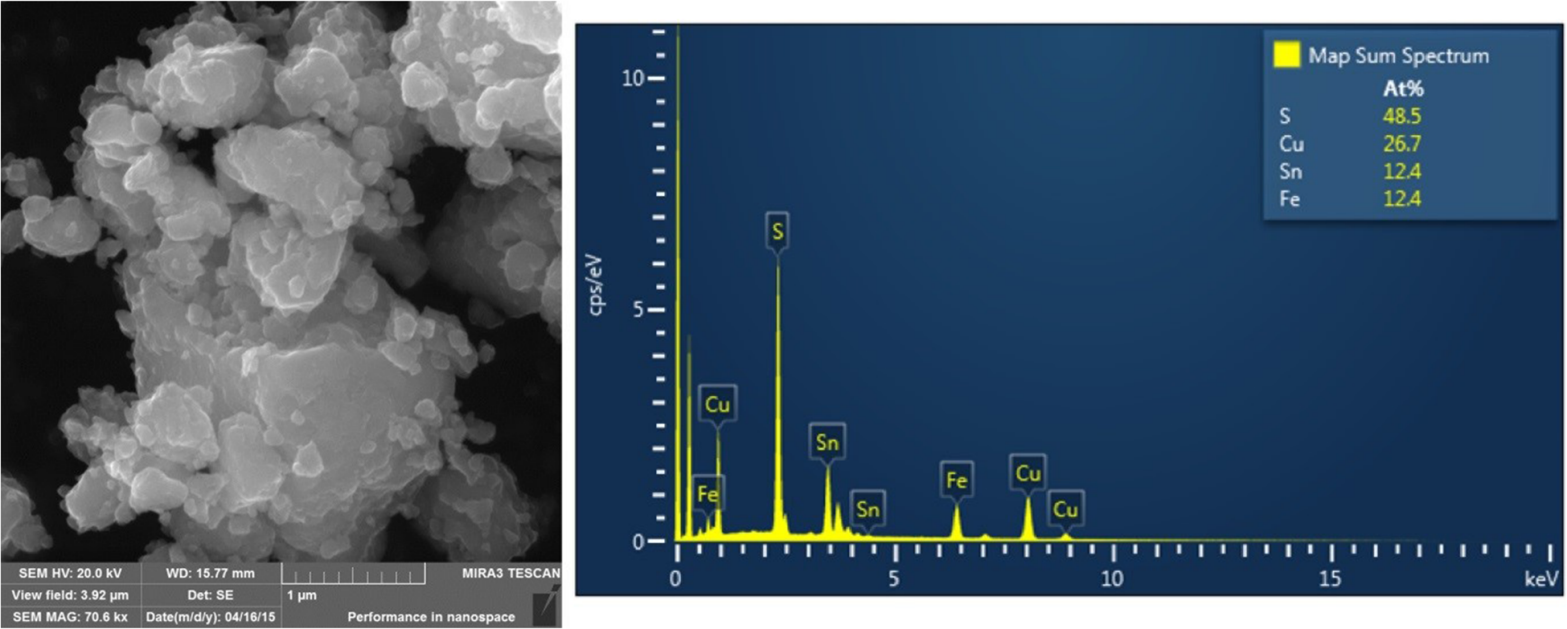
SEM and EDX images of the CFTS sample

**Fig. 6:**
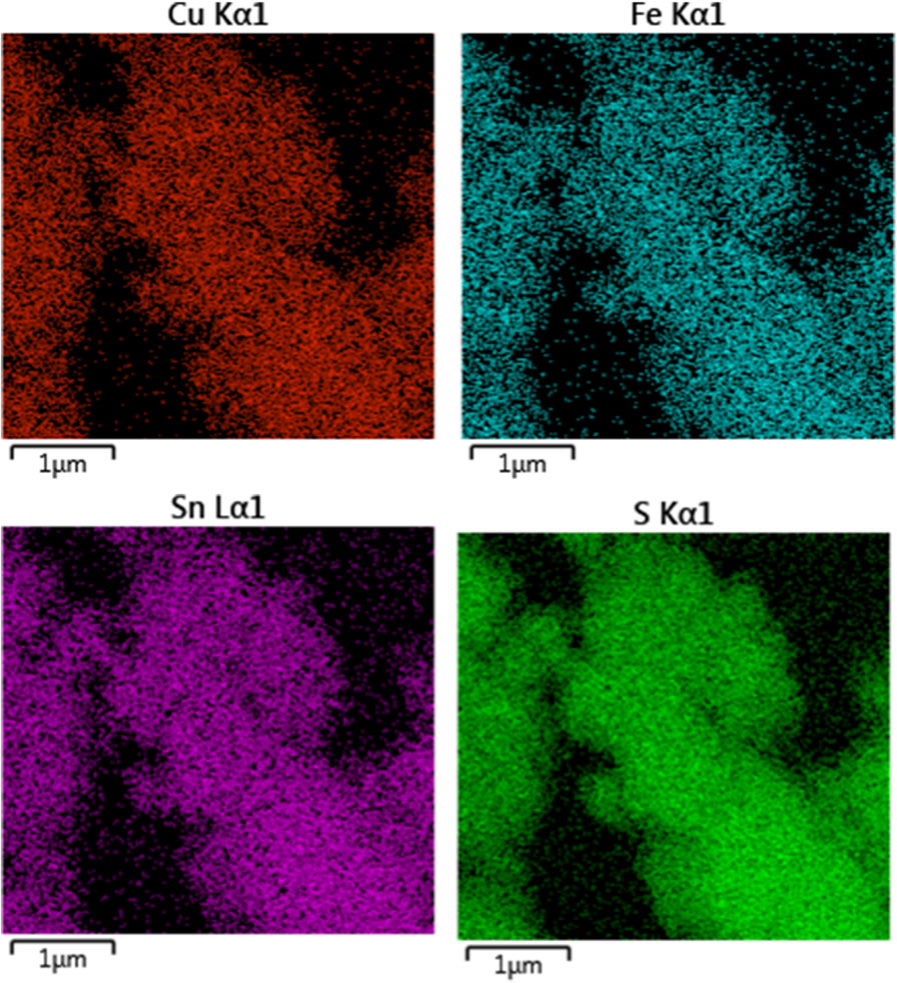
Distribution of Cu, Fe, Sn, and S elements obtained by mapping of the CFTS sample

The results of the SEM and EDX analyses of the CFTS sample together with the elemental mapping are given in Figs. 5 and 6. The SEM image illustrates the clusters of particles in micron and submicron range. The submicron polydisperse aggregates are attached to each other. The number of voids and larger particles are beneficial for photovoltaic applications of polycrystalline materials [14, 39].

The elemental composition of the synthesized CFTS sample was analyzed by EDX spectroscopy. The atomic ratio of the four elements was determined as Cu:Fe:Sn:S = 26.7:12.4:12.4:48.5 (Fig. 5) with the chemical composition Cu_2.15_FeSnS_3.91_, very close to stoichiometry 2:1:1:4. The elemental mapping of the elements documents their uniform distribution in the whole body of the CFTS sample (Fig. 6).

**Fig. 7:**
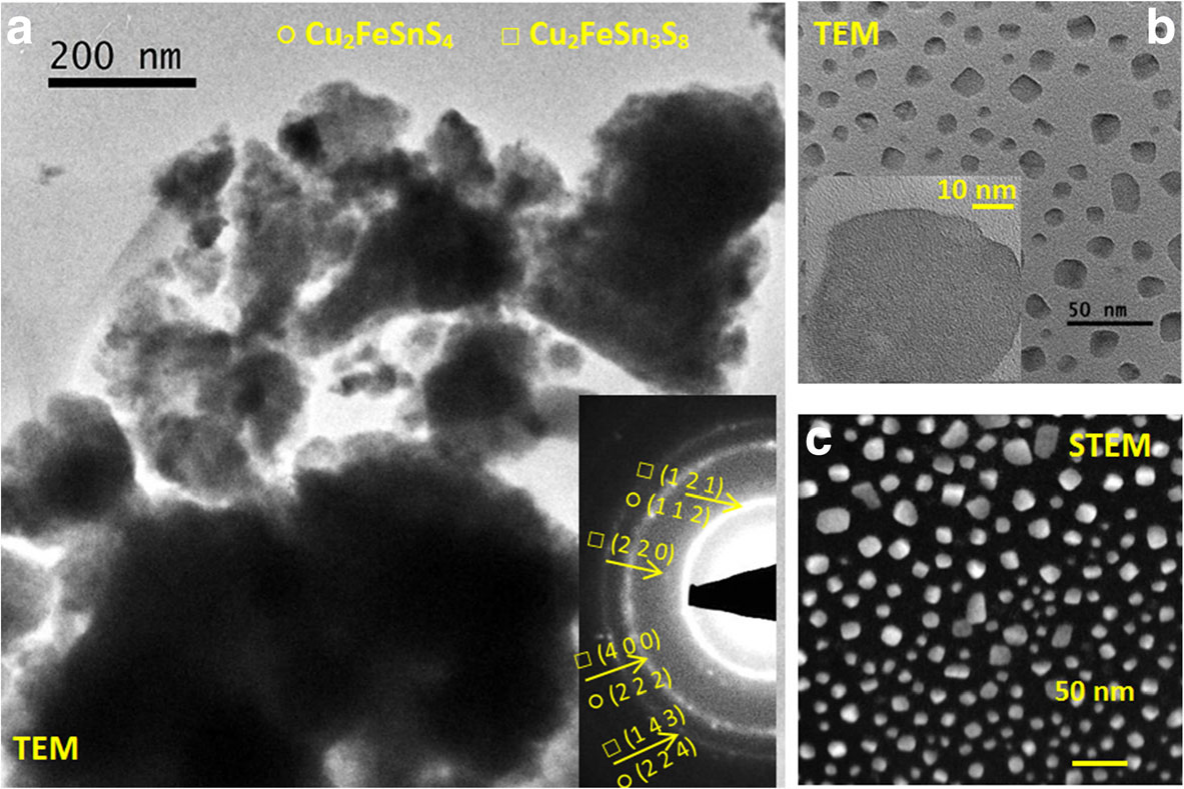
TEM and STEM analysis of the CFTS sample. **a** TEM image of agglomerated particles and the corresponding ED in the *inset*. **b** TEM image of the isolated particles with a particle of about 40 nm in the *inset*. **c** STEM image of the isolated particles

The formation of agglomerates is also illustrated in the TEM micrograph (Fig. 7a). The corresponding EDPs (insets) are formed by rings due to the small coherent diffraction domains (10–20 nm). All rings were indexed in the tetragonal system of the Cu_2_FeSnS_4_ (I-42m space group) and Cu_2_FeSn_3_S_8_ (I41/a space group) compounds, and the planes are marked. These data are in agreement with the X-ray diffraction results presented in Fig. 1 and Table 1. Around the agglomerated particles, very small isolated nanoparticles were found in both samples, as it is shown in the TEM and STEM micrographs presented in Fig. 7b, c. Those nanoparticles show a square-faceted shape with a size between 10 and 40 nm.

**Fig. 8:**
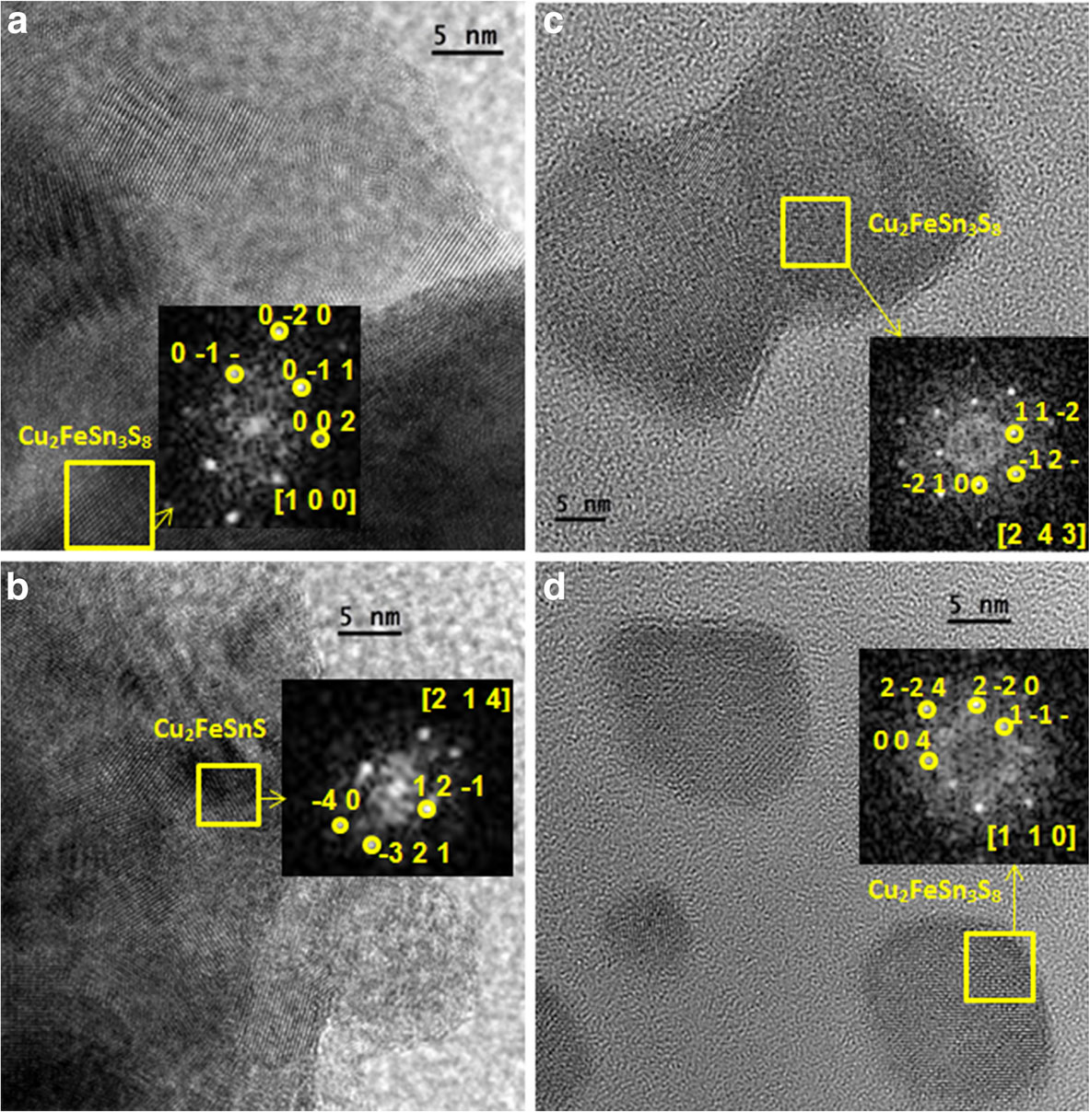
HRTEM images of the CFTS sample. The micrographs show the agglomerated nanocrystals (**a**, **b**) and isolated crystallites (**c**, **d**). The *yellow squares* display oriented crystallites, the corresponding FFT is depicted in the *insets*, and the zone axes and (h, k, l) of the corresponding phase are marked in *yellow*

The HRTEM images of the agglomerated areas show that sample is formed by very small crystallites (Fig. 8a, b). Some oriented crystallites (marked in yellow square) and the corresponding FFT images are depicted in the insets. The analysis of the reciprocal spacings in the FFT allows us to distinguish the particular crystalline phase. The (hkl) planes are marked in the figure. Cu_2_FeSnS_4_ and Cu_2_FeSn_3_S_8_ phases were found in the CFTS sample. As representative examples, a Cu_2_FeSn_3_S_8_ crystallite oriented along the [1 0 0] direction (Fig. 8a) and Cu_2_FeSnS_4_ crystallite oriented in the [2 1 4] zone axis (Fig. 8b) are presented. HR micrographs of the isolated nanoparticles show that many of them are Cu_2_FeSn_3_S_8_ crystals; [2 4 3] and [1 1 0] directions of Cu_2_FeSn_3_S_8_ are presented in Fig. 8c, d, respectively.

**Fig. 9:**
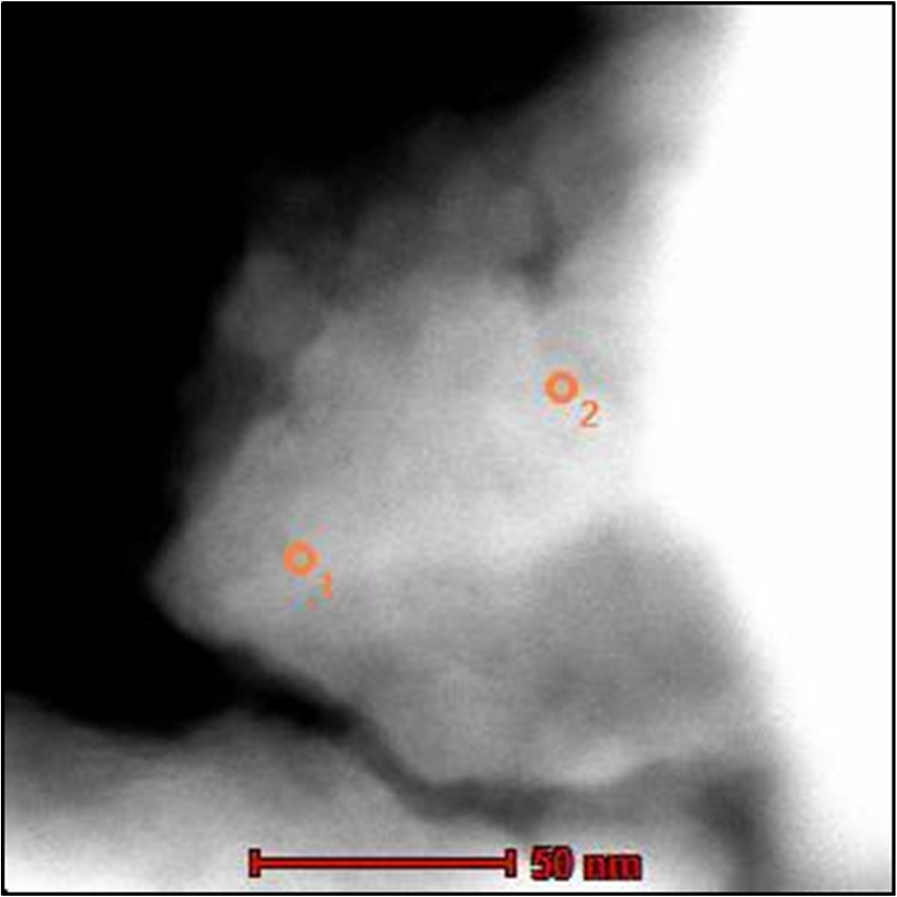
STEM image of the CFTS sample showing the two positions where the EDX analysis was done

The compositional analysis was performed using a combined STEM-EDX technique. The semi-quantitative analysis has shown that the agglomerated areas are formed by both compounds, Cu_2_FeSnS_4_ and Cu_2_FeSn_3_S_8_. In some cases, it was possible to clearly distinguish between the two phases, as can be observed in Fig. 9 and Table 2, where both positions correspond to Cu_2_FeSnS_4_ phase. The results of the EDX analysis, obtained from the TEM microscope, are in accordance with the EDX results when the SEM microscope was used (Fig. 5). The small difference is within a range of the measurement error of the EDX method.

**Table 2:** EDX results of two different positions in the CFTS sample using the STEM microscope

Location	Cu (At %)	Fe (At %)	Sn (At %)	S (At %)
1	25	12.5	12.5	50
2	14.3	7.1	21.4	57.2

### Magnetic Properties

**Fig. 10:**
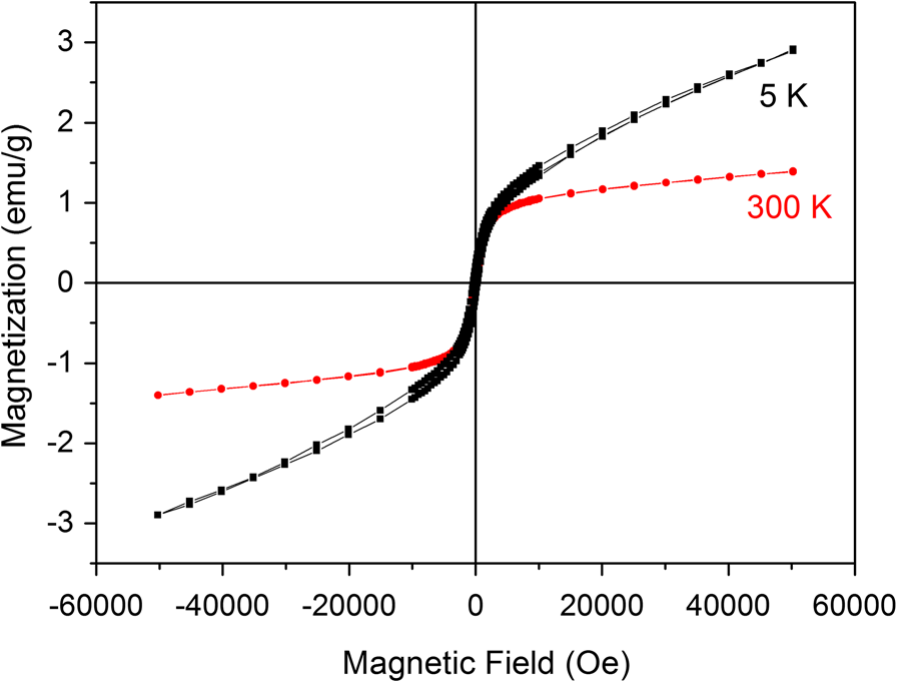
Hysteresis loops of the CFTS sample taken at different temperatures

The hysteresis loops of the mechanochemically synthesized CFTS sample collected at two different temperatures are shown in Fig. 10. The rapid increase of magnetization in the low field part of the hysteresis loop taken at 5 K together with a linear marked increase without a tendency to saturation of its high field part clearly indicates a coexistence of ferromagnetic and paramagnetic phases in the sample. On the other hand, the hysteresis loop taken at 300 K tends to be saturated already in magnetic fields around 10^4^ Oe. This suggests that at a room temperature where the magnetic signals from the paramagnetic Cu_2_FeSnS_4_ and Cu_2_FeSn_3_S_8_ phases are already weak [43, 47], the main contribution to the overall magnetic moment comes from the ferromagnetic component, which is attributed to the elemental Fe, which was not consumed by the mechanochemical reaction. The value of saturation magnetization of iron metal at a room temperature is ~220 emu/g, and therefore, the amount of elemental Fe impurities in our mechanochemically synthesized sample is estimated to be less than 1%.

**Fig. 11:**
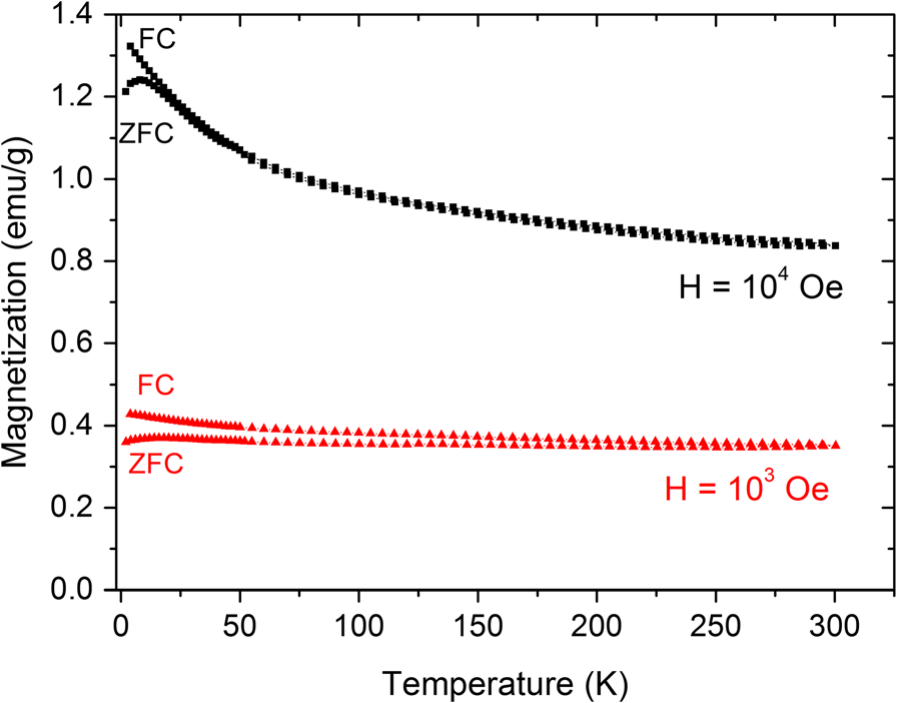
Temperature dependencies of magnetization measured under ZFC and FC conditions at different magnetic field for the CFTS sample

Figure 11 shows the temperature dependencies of magnetization measured in applied fields of 10^3^ and 10^4^ Oe under ZFC and FC conditions. The CFTS sample is composed mostly of quaternary tetragonal polymorphs of stannite Cu_2_FeSnS_4_ and rhodostannite Cu_2_FeSn_3_S_8_ with relatively weak magnetic moments, but it includes also a minor amount of ferromagnetic Fe impurities, which exhibit a strong response to the applied magnetic field. This makes the analysis of ZFC and FC curves rather complicated, because the overall magnetic signal is a sum of individual components exhibiting different variation of magnetization with temperature. The paramagnetic Cu_2_FeSnS_4_ was reported to show a transition to the antiferromagnetic behavior below 38 K [43]. More recently, Cui et al. reported that Cu_2_FeSnS_4_ nanocrystals show a weak ferromagnetic behavior at cryogenic temperatures and a paramagnetic response at higher temperatures [13]. Paramagnetic Cu_2_FeSn_3_S_8_ exhibits an interesting “high spin–low spin transition”, which is of the continuous type and extends over a very broad temperature range [47]. As can be seen in Fig. 11, the field of 10^3^ Oe corresponds to the low field part of hysteresis loop where the magnetization of non-consumed elemental Fe with high Curie temperature dominates over the magnetic contributions from paramagnetic and/or other weak magnetic phases in CFTS sample. Hence, ZFC and FC curves taken under the application of this low applied field are relatively featureless and exhibit weak temperature dependence. An increase of measurement field to 10^4^ Oe results in more pronounced changes of magnetization with temperature, which is attributed to the more significant contribution of Cu_2_FeSnS_4_ and Cu_2_FeSn_3_S_8_ phases to the overall magnetic moment after application of high magnetic field. A clear splitting between ZFC and FC curves below 18 K is very similar to that observed for Cu_2_FeSnS_4_ nanocrystals in [13]. Therefore, a plausible explanation of the marked low-temperature downturn of ZFC branch is the transition from paramagnetic to weak ferromagnetic behavior of Cu_2_FeSnS_4_ phase in the CFTS sample.

## Conclusions

We have demonstrated the simple one-pot mechanochemical synthesis of CFTS nanocrystals with the properties suitable for the applications in solar cell materials. Stannite Cu_2_FeSnS_4_ and rhodostannite Cu_2_FeSn_3_S_8_ were identified by the XRD method. The calculated crystallite sizes for both phases were 18–19 nm. The resulting CFTS nanocrystals exhibit a bandgap 1.25 eV, which is very well-acceptable value for the application as a photo-absorber in solar cell materials. The bandgap energy can be manipulated by milling. Raman spectra, SEM, HRTEM, and EDX analyses confirmed the successful formation of CFTS. Using SQUID magnetometry, weak ferromagnetic properties of the synthesized nanocrystals were documented. The applied mechanochemical approach represents simple, solvent-free reproducible fabrication of CFTS which might be easily scaled up. However, there remains a challenge of the separation of rhodostannite and stannite phases, which could be a possible motivation for future research in this field. Mechanochemistry seems to be a proper tool to manipulate with the distribution of both phases.

## Abbreviations

CFTS: Cu_2_FeSnS_4_
CIGS: CuIn_1 − *x*_Ga_*x*_Se_2_
CZTS: Cu_2_ZnSnS_4_
EDX: Energy-dispersive X-ray
FC: Field-cooled
FFT: Fast Fourier transform
HAADF: High-angle annular dark field
HRTEM: High-resolution transmission electron microscopy
SEM: Scanning electron microscopy
ZFC: Zero-field-cooled
